# Xenon Pretreatment May Prevent Early Memory Decline after Isoflurane Anesthesia and Surgery in Mice

**DOI:** 10.1371/journal.pone.0026394

**Published:** 2011-11-03

**Authors:** Marcela P. Vizcaychipi, Dafydd G. Lloyd, Yanjie Wan, Mark G. Palazzo, Mervyn Maze, Daqing Ma

**Affiliations:** 1 Anaesthetics, Pain Medicine and Intensive Care, Department of Surgery and Cancer, Imperial College London, Chelsea and Westminster Hospital, London, United Kingdom; 2 Department of Anesthesiology, Gongli Hospital, Shanghai, China; 3 Department of Anesthesia and Perioperative Care, University of California San Francisco, San Francisco, California, United States of America; Centre de Recherche Public de la Santé (CRP-Santé), Luxembourg

## Abstract

Postoperative cognitive decline (POCD) is a common complication following surgery, but its aetiology remains unclear. We hypothesized that xenon pretreatment prevents POCD by suppressing the systemic inflammatory response or through an associated protective signaling pathway involving heat shock protein 72 (Hsp72) and PI3-kinase. Twenty-four hours after establishing long-term memory using fear conditioning training, C57BL/6 adult male mice (n = 12/group) received one of the following treatments: 1) no treatment group (control); 2) 1.8% isoflurane anesthesia; 3) 70% xenon anesthesia; 4) 1.8% isoflurane anesthesia with surgery of the right hind leg tibia that was pinned and fractured; or 5) pretreatment with 70% xenon for 20 minutes followed immediately by 1.8% isoflurane anesthesia with the surgery described above. Assessments of hippocampal-dependent memory were performed on days 1 and 7 after treatment. Hsp72 and PI3-kinase in hippocampus, and plasma IL-1β, were measured using western blotting and ELISA respectively, from different cohorts on day 1 after surgery. Isoflurane induced memory deficit after surgery was attenuated by xenon pretreatment. Xenon pretreatment prevented the memory deficit typically seen on day 1 (P = 0.04) but not on day 7 (P = 0.69) after surgery under isoflurane anesthesia, when compared with animals that underwent surgery without pretreatment. Xenon pretreatment modulated the expression of Hsp72 (P = 0.054) but had no significant effect on PI3-kinase (P = 0.54), when compared to control. Xenon pretreatment also reduced the plasma level increase of IL-1β induced by surgery (P = 0.028). Our data indicated that surgery and/or Isoflurane induced memory deficit was attenuated by xenon pretreatment. This was associated with a reduction in the plasma level of IL-1β and an upregulation of Hsp72 in the hippocampus.

## Introduction

Postoperative cognitive decline (POCD) is a frequent complication of major surgery in the elderly [1], with an incidence of up to 56% [2]. However, the pathogenesis and molecular mechanisms underlying POCD have so far remained unclear. Risk factors for POCD include increasing age, preoperative cognitive dysfunction and perioperative infection [2], [3], [4]. Central inflammatory responses, specifically cytokine increases in the hippocampus following surgery, have been reported in rat and mouse models of POCD [5], [6]. Increase in hippocampal IL-1β is suggested to play a role in the onset of a memory deficit following infection [7], [8], [9]. Furthermore, the possible contributory role of anesthesia and analgesia in the development of POCD remains ill defined [10].

Physiologically protective mechanisms may prevent or alter neuroinflammatory changes and could influence the development of POCD [11], [12]. For example, cytosolic heat shock protein 72 (Hsp72) is readily induced by stress and has cellular protective functions [13]. Studies of *in vitro* and *in vivo* models of stroke have demonstrated that injury can be reduced by Hsp72 over-expression [11]. Hsp72 over expression was also found to be associated with a reduced activation of the pro-inflammatory transcription factor NF-kB [14]. Our previous study showed that transgenic over expression of Hsp72 was associated with better preservation of memory following tibial fracture surgery [15]. Anesthetics including xenon and isoflurane also have effects on cellular signaling that may mediate their organoprotective properties [16].

Xenon has been found to confer neuroprotection in animal models of cerebral ischemia [17], cardiopulmonary bypass [18], [19], and post-cardiac arrest [20]. One mechanism by which xenon exerts such effects is by inducing hypoxia inducible factor one alpha, (HIF-1α) [17] which can interfere with apoptotic signaling pathways [21], [22]. To our knowledge, there are no data in the literature that describe the effects of xenon on stress proteins, including Hsp72, or its role in the prevention of postoperative memory decline.

The aim of our current study was to investigate whether xenon pretreatment could prevent or reduce memory impairment following surgery in mice. We hypothesized that xenon pretreatment would have such an effect due to a reduction in the systemic inflammation associated with upregulation of cytoplasmic Hsp72, via the PI3-kinase pathway.

## Results

**Figure 1:**
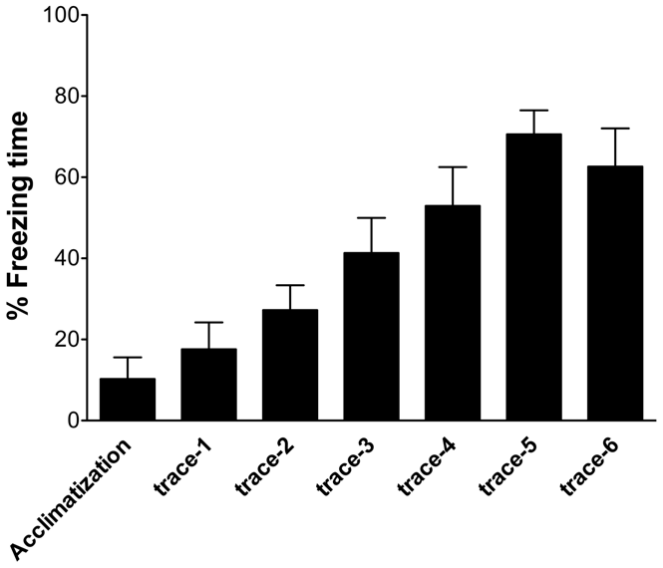
Learning and memory retrieval using fear conditioning paradigm. Memory acquisition was achieved by trial 4 and asymptotic performance was achieved by trial 6 during a single training session.

All animals were trained prior to treatment. Memory acquisition by association was achieved by trial 4 and consolidated by trial 6 of paired conditional - unconditional stimuli (CS - US) during a single training session. Asymptotic performance was achieved by the end of the sixth pair of CS – US stimuli (Figure 1). The mean % freezing time during the acclimatization period was 10±5% increasing to 63±9% by the sixth pair of stimuli. There were no statistically significant differences between the experimental animal groups prior to surgery in either acquisition or memory formation during the conditional response, trace interval, unconditional response and inter-pair intervals during training (data not shown).

### Effects of Surgery on Behavior

**Figure 2:**
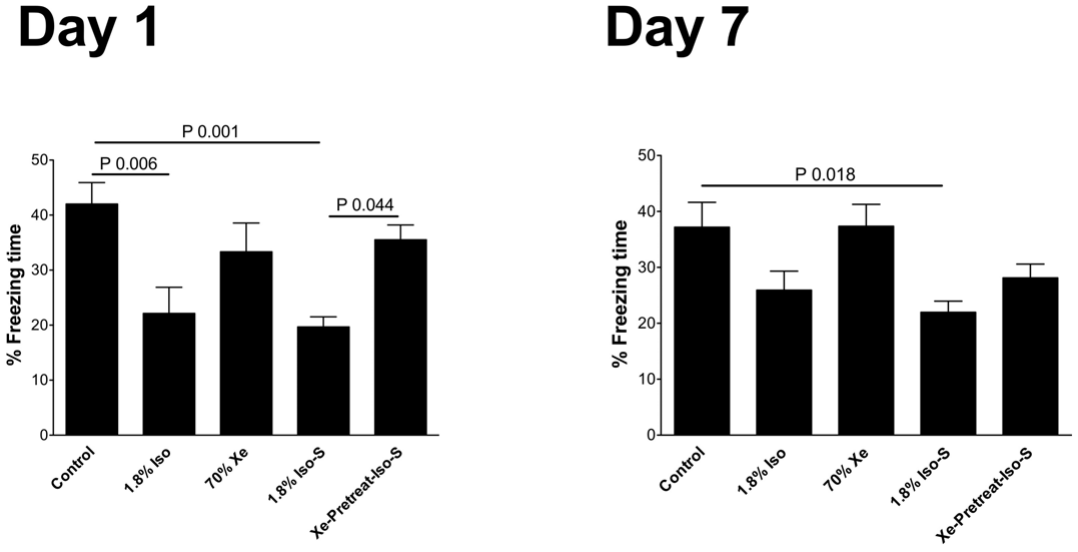
Hippocampal dependent memory is impaired following treatment. A contextual conditional response, which is a form of hippocampal dependent memory, was assessed as the percentage freezing time on days 1 (n = 12/group) and 7 (n = 12/group) after treatment. The experimental animal groups are as described in the methods. Values are the mean ± SEM. P values as indicated in the figures.

The surgery under isoflurane anesthesia group showed reduced memory on day 1 when compare with control, P = 0.001 (Table 1). Mice preconditioned with xenon prior to surgery did not show significant memory impairment on day 1. Their memory retention was significantly better than the surgical group with no pretreatment, P = 0.044 (Figure 2 and Table 1).

**Table 1:** Hippocampal dependent memory: Contextual conditional response was assessed by % freezing time.

	Control	1.8% Iso	70% Xe	1.8% Iso-S	Xe-Pretreat-Iso-S
Day 1	42.0±3.8%	22.1±4.7%	33.3±5.2%	^§^19.6±1.8%	^§§^32.1±2.6%
Day 7	37.1±4.4%	25.9±3.3%	37.3±3.9%	^□^21.9±1.9%	28.1±2.4%

Data is presented as mean ± SEM. Day 1: ^§^ P = 0.001 compared to control, ^§§^P = 0.044 compared to Iso-S. Day 7: ^□^P = 0.018 compared to control.

*1.8% Iso = 1.8% isoflurane anesthesia treatment, 70% Xe = 70% xenon treatment, 1.8% Iso-S = 1.8% isoflurane surgery treatment, Xe-Pretreat-Iso-S = xenon pretreatment +1.8% isoflurane anesthesia for surgery group.*

By day 7, there was no difference in retrieval of hippocampal dependent memory between the two surgical groups, P = 0.69. However, the surgical group that had not received xenon pretreatment exhibited reduced memory retention as compared to control, P = 0.018 (Figure 2 and Table 1).

### Hippocampal-independent memory

Freezing behavior during the conditional stimulus assesses hippocampal independent memory involving brain regions including the prefrontal cortex and amygdala. We assessed the dependence of the non-conditional response (tone) to treatments on days 1 and 7. We found that surgery under isoflurane anesthesia caused a reduction in freezing time on day 1 as compared to control, P = 0.034. By day 7, animals showed memory decline when compared to control: 1.8% Iso (P = 0.012), 1.8% Iso-S (P = 0.052) and Xe-Iso-S (P = 0.017).

The variability in hippocampal dependent memory was minimal compared to that of hippocampal independent memory, where more inter-animal variability was seen within the groups (Table 2).

**Table 2:** Hippocampal independent memory: Conditional response was assessed by.

	Control	1.8% Iso	70% Xe	1.8% Iso-S	Xe-Pretreat-Iso-S
Day 1	56.8±6.3%	^§^30.8±8.4%	^§§^28.3±7.4%	^§§§^28.9±5.9%	34.5±4.0%
Day 7	43.1±4.6%	^□^19.6±4.5%	32.1±6.3%	^□□^13.8±2.2%	^□□□^15.5±2.6%

Data is presented as mean ± SEM. Day 1: ^§^P = 0.57, ^§§^P = 0.029, ^§§§^P = 0.034, Day 7: ^□^P = 0.012, ^□□^P = 0.052, ^□□□^P = 0.017, all compared to control.

*1.8% Iso = 1.8% isoflurane anesthesia treatment, 70% Xe = 70% xenon treatment, 1.8% Iso-S = 1.8% isoflurane surgery treatment, Xe-Pretreat-Iso-S = xenon pretreatment +1.8% isoflurane anesthesia for surgery group.*

### PI3-kinase and Hsp72 analysis

**Figure 3:**
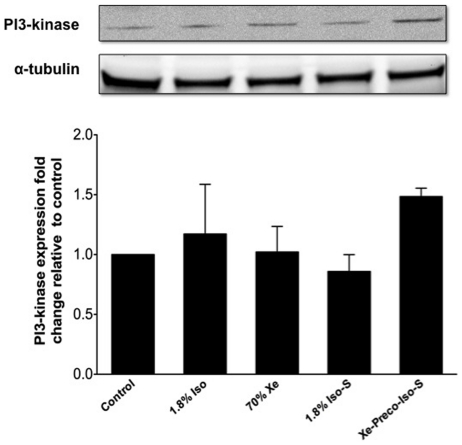
Western blotting analysis of PI3-kinase in mouse hippocampal tissue on day 1 after treatment. The experimental animal groups are as described in the methods (n = 4/group). The top panel shows a representative immunoblot for PI3-kinase that was reprobed for α-tubulin as a loading control. The histogram in the bottom panel shows the densitometric measurements of the PI3-kinase signals on the immunoblot, relative to control. Values are the mean ± SEM.

Hippocampi of 4 mice out of 6 per group were analyzed for PI3-kinase on day 1 after treatment. Tissue from 2 animals per group were not analyzed due to technical problems with western blotting membrane. There was greater expression of PI3-kinase expression on day 1 in mice exposed to xenon pretreatment prior to surgery as compared to the control group, but this did not reach to a statistical significance (P = 0.54) (Figure 3).

**Figure 4:**
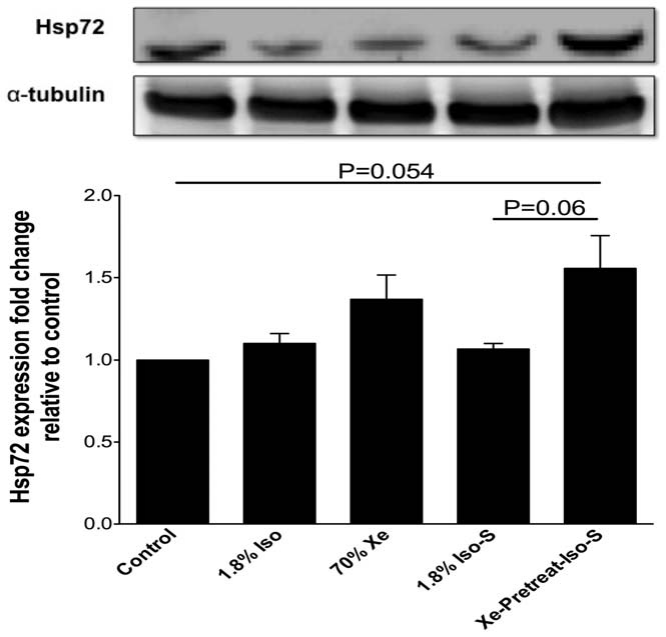
Western blotting analysis of Hsp72 in mouse hippocampal tissue on day 1 after treatment. The experimental animal groups are as described in the methods (n = 6/group). The top panel shows a representative immunoblot for Hsp72 that was reprobed for α-tubulin as a loading control. The histogram in the bottom panel shows the densitometric measurements of the Hsp72 signals on the immunoblot, relative to control. Values are the mean ± SEM.

Hippocampi from 6 mice/group were assessed for Hsp72. The Hsp72 expression was elevated in the Xe-pretreat-Iso-S group on day 1 after treatment. Expression of Hsp72 in the hippocampi of Xe-pretreat-Iso-S group showed a positive modulation compared to that in the control group (1.7±0.2) (P = 0.054). There was also difference between Xe-pretreat-Iso-S and 1.8% Iso-S on day 1 after treatment (P = 0.06) (Figure 4).

### IL-1β analysis

**Figure 5:**
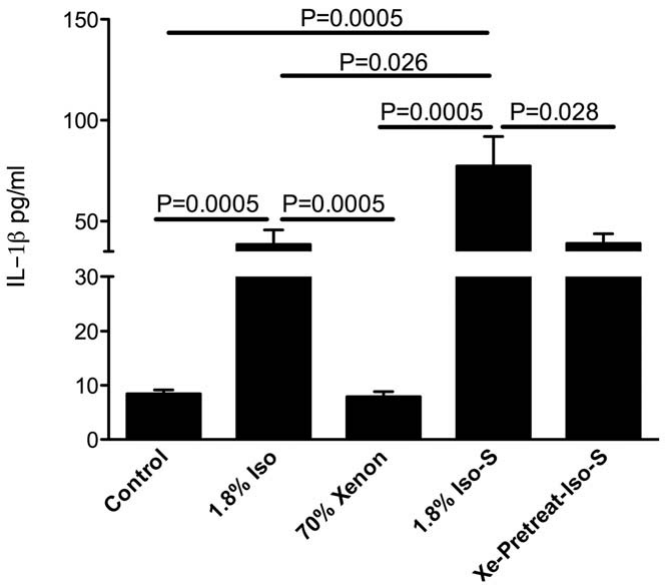
ELISA of plasma IL-1β levels on day 1 after treatment. The experimental animal groups are as described in the methods (n = 4/group). Values are the mean ± SEM.

Plasma IL-1β levels were measured on day 1 after treatment. IL-1β plasma levels were 8.43±0.7 pg/ml in the control, 41.5±6.8 pg/ml in the Xe-pretreat-Iso-S (P = 0.137) and 77.3±14.6 pg/ml in the 1.8% Iso-S groups (P = 0.0005). There was a significant difference between the Xe-pretreat-Iso-S and 1.8% Iso-S groups (P = 0.028). The 1.8% Iso-S anesthesia group also had significant difference in plasma level of IL-1β when compared to 70% Xe anesthesia and control groups, on day 1 after treatment (P = 0.0005), and 1.8% Iso (P = 0.026) (Figure 5).

## Discussion

The results of our current study suggest that early postoperative memory decline after surgery under isoflurane anesthesia is ameliorated by xenon exposure immediately prior to surgical intervention. This is associated with an upregulation of Hsp72 expression and PI3-kinase in the hippocampus and a decrease in the plasma levels of IL-1β. Our data also suggest that the detrimental effect is due to anesthesia in our animal model, which is rescued by pretreatment of the animal with xenon.

We previously reported that no memory decline was seen after tibial fracture surgery under general isoflurane anesthesia in Hsp72-overexpressing transgenic mice [15]. Thermal and chemical-induced Hsp72 preconditioning in a rat heatstroke model has been shown to have protective effects that correlate with specific anatomical and histological changes in the brain [23]. In line with our study, previous work has shown that upregulation of Hsp72 prior to myocardial ischemia confers cardioprotection as reflected by reduced markers of cellular damage and improved functional recovery [24]. Short periods of xenon treatment have been clearly shown to upregulate several pro-survival proteins including Bcl-2 [25], [26], and HIF-1α [17], [27]. Our data suggest that the increase in expression of Hsp72 24 hours after exposure to xenon might be an important molecular mechanism by which xenon prevents memory decline in the early postoperative period. Moreover, the attenuation of cognitive decline following surgery through xenon pretreatment is also likely to be due to its activation of the PI3-kinase pathway.

Xenon pretreatment was shown to inhibit the elevation of cytokine IL-1β in plasma after surgery. Xenon pretreatment may only have effect on proteins already synthesized or stored, ready to be released or expressed in the event of an insult, as a response to stress. Preconditioning may be considered to prepare the cells for a major insult by modifying protein synthesis. Consistent with our observation, a clinical study showed that xenon seems to confer protective effects by inhibition of platelets and leucocytes activation during cardiopulmonary bypass in humans [28]. It has also been shown that xenon has anti-inflammatory effects including reducing plasma levels of tumor necrosis factor-1 alpha (TNF-1α) and interleukin-6 (IL-6) after myocardial injury in a porcine model of right ventricular infarction [29]. This anti-inflammatory effect of xenon is likely to contribute to its beneficial effects. The neuroinflammatory response is likely to be a key mechanism in cognitive decline after surgery, as demonstrated clearly by our group and others [5], [6], [30]. Recent work in immortalized microglia stimulated with lypopolyssacharide (LPS) found a pro-inflammatory effect of xenon [31]. The reason for these discrepancies remains unclear. However, there are species, stress and cell type differences between these studies and ours.

It should be pointed out that the attenuating effect of xenon on early postoperative cognitive decline may also be modulated by isoflurane anesthesia. This is because, like xenon, isoflurane may have some anti-inflammatory effects. Previous studies showed that pretreatment with isoflurane reduced microglial activation at 24 hours after LPS plus interferon gamma exposure in C8-B4 microglia [32]. Another inhalational anesthetic, sevoflurane, induced an increase in expression of Hsp72 in cardiac muscle [33]. We were unable to demonstrate similar changes with the administration of isoflurane in terms of Hsp72 expression and lower level of IL-1β in plasma. This could be secondary to inter-species variability between mice and rabbits [33]. Nevertheless, isoflurane anesthesia has been considered to be a risk factor for cellular toxicity and cognitive decline as reported previously *in vitro* [34] and *in vivo* [35], [36], [37] respectively or to induce proinflammatory cytokine release in mice. (Wu et al., 2010)

Three limitations to our study should be noted: 1) A group of animals pretreated with isoflurane followed by surgery or even a group treated with xenon followed by isoflurane alone would contribute to the understanding of our memory assessment findings. 2) The surgical model used in our study is a very specific iatrogenic injury generated under general anesthesia where other variables were tightly controlled and which may not closely mimic the clinical situation; 3) Adult rather than aged mice were used in the assessment of postoperative memory decline after surgery while POCD is more often to be seen in elderly patients. Moreover, our tibial fracture model may not trigger a categorical or pronounced behavioral change to those seen after exposure to isoflurane alone. Also fracture trauma generated under general anesthesia may not represent the insult seen in humans after trauma. However, in view of the significant difference in behavior seen after exposure to xenon, we consider it important to alert our community about the potential risk or influence of anesthesia on cognition after surgery. Despite these limitations, we did observe a protective effect of xenon pretreatment on early postoperative memory decline.

### Conclusion

Our present data demonstrate that xenon exposure immediately prior to surgery prevents early memory decline in mice, as assessed by fear conditioning after tibial fracture surgery. This is associated with a positive modulation of Hsp72 expression in the hippocampus and a reduction in the plasma level of IL-1β. Our present results thus raise the possibility that xenon anesthesia may reduce the risk of early cognitive impairment following surgery.

## Materials and Methods

The study was approved by the UK Home Office and conforms to the United Kingdom Animals (Scientific Procedures) Act of 1986, PPL 70/6966, 19b 10.

### Animals

C57BL/6 male mice (Harlan, Bicester, Oxon, UK) (14±2 weeks old, weighing 25–30 g) were used in this study and were housed in groups of five in a 12-h-12-h light dark cycle environment in which temperature and humidity were controlled. The mice were allowed free access to food and water. The animal holding and experimental room was maintained with temperature at 20–22°C and humidity at 35–55%.

### Conditional memory formation training

In our study, fear conditioning behavioral test paradigm has been used to assess conditioned memory formation in the hippocampus in our animal model. We consider this to be analogous to common tests of memory utilized to assess memory decline after surgery in humans. One day prior to treatment, the animals were trained in fear conditioning to establish long-term memory. Training and testing were performed in chambers with a video camera positioned on one of the lateral walls to allow the subjects' behavior to be observed and recorded by an investigator, both on- and off-line. The floor consisted of 32 stainless steel rods (1 mm diameter), spaced 0.5 cm apart (center-to-center), and wired to a shock generator and scrambler that delivered a 0.70 mA foot shock.

Training and testing for fear to the context took place in two identical conditioning chambers (17.78×19.05×38.10 cm) housed in sound attenuating box (Med Associates Inc, St Albans, VT). The chambers were cleaned with a 5% sodium hydroxide solution scented with 0.2% mint extract. Memory was established in the preoperative period by exposing animals to the fear conditioning chamber with background noise (60 dB) provided by a fan positioned behind the chamber. Animals were acclimatized for 120 seconds before exposure to the training session that consisted of six trials of paired conditional stimuli (tone, for 30 seconds at 90 dB) and unconditional stimuli (shock, for 2 seconds at 0.70 mA). Trace interval was 30 seconds between tone and shock, with pseudorandom inter-training intervals of between 30 and 45 seconds. The total training time for each animal was 667 seconds. Hippocampal dependent memory and hippocampal independent memory were assessed in a different context from which the animals were trained.

Freezing was defined as the absence of any visible movement, except that required for respiration (only fluctuation in the volume of the thorax). The threshold for freezing was chosen according to the individual animals motion index histogram, analyses being performed by Video Freeze Version 2.1.0 (Med Associates Inc, St Albans, VT). Recording starting 20 seconds after placing the animals in the chamber, with a bout length of 0.75 seconds used in the algorithm to identify episodes of freezing by the software. The percentage of freezing-time, an indicator of memory formation, was assessed by an independent operator for further statistical analysis using the video recordings and tabulated data generated by the software.

### Contextual memory assessment

Assessments of contextual memory formation and conditional responses to test hippocampal dependent and independent memory were performed in a novel context on days 1 and 7 after treatment, in a different cohort of animals for each corresponding day [38]. The percentage of the total time spent freezing (% freezing time) was used as an indicator of memory formation during training and memory retrieval after surgery. Hippocampal dependent memory was assessed by total freezing time during exposure to a novel context [39], [40] while hippocampal independent memory was assessed by the percentage of freezing time during exposure to the conditional stimulus (tone).

### Anesthesia and surgery

Mice were allocated to 1 of 5 experimental groups (n = 12 mice/group) undergoing one of the following treatments 24 hours after training: 1) control - no treatment (Control); 2) 1.8% isoflurane anesthesia for 20 minutes (1.8% Iso); 3) 70% xenon anesthesia balanced with nitrogen for 20 minutes (70% Xe); 4) 1.8% isoflurane anesthesia with surgery of the right hind leg tibia that was pinned and fractured (1.8% Iso-S); and 5) pretreatment with 70% xenon for 20 minutes followed immediately by 1.8% isoflurane anesthesia with surgery (Xe-pretreat-Iso-S). The duration of surgery under anesthesia was approximately 20 minutes in all cases. The anesthesia consisted of 1.8% isoflurane in 30% oxygen balanced with nitrogen [41]. The concentrations of isoflurane or xenon were monitored with a Datex Ohmeda (GE Healthcare, Bradford, UK) and xenon monitor (Air product, Oxon, UK), respectively. The surgical model adopted in this study is based on that described previously [42]. Briefly, under monitored isoflurane anesthesia, skin incision was made below the knee and soft tissue was reflected to expose bone through which a 0.3 mm stainless steel rod was inserted into the medullary cavity. Once the tibia was internally fixed, the bone was fractured in the mid-diaphysis (tibia, mid-shaft) using surgical pliers. The wound was then closed using 2-0 Nylon sutures (Ethicon Inc, Somerville, NJ). A needle thermistor was inserted into the subcutaneous tissue of the left leg. Temperature was maintained at 37.0±0.4°C throughout these experiments using a servo-controlled heating lamp and heating pad. Mice were allowed to recover on heat pads and then transferred to their own cages where food and water were available. Post-surgery analgesia was provided using intraperitoneal (i.p.) buprenorphine, 0.03 mg/kg in saline and topical eutectic mixture of local anesthesia (EMLA) twice daily for 72 hours.

### Western blotting analysis

Both hippocampi from additional mouse cohorts (n = 6/group) treated as described, but without behavioral testing, were harvested 24 hours after treatment and homogenized (Polytron homogenizer by Kinematrica, Bethlehem, PA) in iced- cold cell lysis buffer (20 mM Tris-HCL, 150 mM NaCL, 1 mM Na2EDTA, 1 mM EGTA, 1% Triton, 2.5 mM sodium pyrophosphate, 1 mM β-glycerolphosphate, 1 mM Na3VO4, 2 mM DL-dithiothreitol, 1 mM phenylmethanesulphonyl , and 1 µg/ml leupeptin, pH 7.5). The homogenates were centrifuged at 14,000 rpm for 10 minutes and the supernatants used for western blotting after protein quantification. Each sample of 20 µg protein in NuPage® SDS sample buffer (Invitrogen, Carlsbad, CA) was separated by sodium dodecyl sulfate-polyacrylamide gel electrophoresis (SDS-PAGE; Invitrogen) and transferred to a nitrocellulose membrane (Hybond ECL, Amersham Biosciences, Little Chalfont, UK). The membranes were blocked for 3 h in 5% non-fat powdered milk in Tris-buffered saline/Tween (50 mM Tris base, 150 mM NaCl, 0.1% Tween 20, pH 7.4) and then incubated with Hsp70 (Hsp72) monoclonal antibody (C92F3A-5) biotin conjugate (1∶1000 dilution) (Stressgen assay designs, catalog number: SPA-810B) or PI3-kinase p110 gamma antibody (1∶1000 dilution) (Cell Signaling; Ref: 4252), overnight at 4°C. This was followed by incubation with secondary horseradish peroxidase-conjugated antibody. Protein bands were visualized using an enhanced chemiluminescence system (Cell Signaling Technology, Hertfordshire, UK). The membranes were then stripped and re-incubated with monoclonal-anti-α-tubulin as a loading control (1∶1000 dilution). Protein bands were captured using the image processor GeneSnap (Syngene, Cambridge, UK). Their density, measured with GeneTools software (Syngene, Cambridge, UK), was normalized using α-tubulin signals and expressed as ratio of the control.

### ELISA

Blood was sampled transcardially after thoracotomy under terminal isoflurane anesthesia in a separate animal cohort that received the same treatments as the behavior-assessed groups. Blood samples taken from treatment free animals served as controls. Samples were centrifuged at 3,600 rpm for 30 minutes at 4°C and stored at −80°C prior to analysis. IL-1β was measured using commercially available ELISA kits from Bender MedSystems® (Ref: BMS6002). The sensitivity of the assay was 1.2 pg/ml.

### Statistical analysis

Data are expressed as the mean ± SEM. All data were analyzed using one-way analysis of variance (ANOVA) followed by Tukey's multiple comparison test to compare all groups (Prism 5 for Mac OS X, Software, Inc). A Tukey multiple comparison test P value<0.05 was considered statistically significant.
